# Crystallography of homophase twisted bilayers: coincidence, union lattices and space groups

**DOI:** 10.1107/S2053273323003662

**Published:** 2023-06-02

**Authors:** Denis Gratias, Marianne Quiquandon

**Affiliations:** a CNRS UMR 8247, Institut de Recherche de Chimie ParisTech, 11 rue Pierre et Marie Curie, 75005 Paris, France; Ateneo de Manila University, Philippines

**Keywords:** bicrystallography with complex numbers, bilayers, coincidence lattices, space groups

## Abstract

This paper presents the basic tools used to describe the global symmetry of so-called bilayer structures obtained when two differently oriented crystalline monoatomic layers of the same structure are superimposed and displaced with respect to each other. The 2D nature of the layers leads to the use of complex numbers that allows for simple explicit analytical expressions of the symmetry properties involved in standard bicrystallography [Gratias & Portier (1982). *J. Phys. Colloq.* **43**, C6-15–C6-24; Pond & Vlachavas (1983). *Proc. R. Soc. Lond. Ser. A*, **386**, 95–143]. The focus here is on the twist rotations such that the superimposition of the two layers generates a coincidence lattice. The set of such coincidence rotations plotted as a function of the lengths of their coincidence lattice unit-cell nodes exhibits remarkable arithmetic properties. The second part of the paper is devoted to determination of the space groups of the bilayers as a function of the rigid-body translation associated with the coincidence rotation. These general results are exemplified with a detailed study of graphene bilayers, showing that the possible symmetries of graphene bilayers with a coincidence lattice, whatever the rotation and the rigid-body translation, are distributed in only six distinct types of space groups. The appendix discusses some generalized cases of heterophase bilayers with coincidence lattices due to specific lattice constant ratios, and mechanical deformation by elongation and shear of a layer on top of an undeformed one.

## Abstract 2

A general scheme is proposed to classify and determine the crystallographic properties of twisted bilayers of any homophase 2D structures using complex numbers.

## 1. Introduction

The discovery of strong electronic correlations and superconductivity in twisted bilayer graphene (Trambly de Laissardière *et al.*, 2010 ▸, 2012 ▸), with a so-called magic rotation angle close to 1.05° where the Fermi velocity vanishes, has significantly increased the interest in detailed study (Cao, Fatemi, Demir *et al.*, 2018 ▸; Cao, Fatemi, Fang *et al.*, 2018 ▸) of these kinds of low-dimension structures [see, for transition metal dichalcogenides, Naik & Jain (2018 ▸), Wu *et al.* (2019 ▸), Soriano & Lado (2020 ▸), Venkateswarlu *et al.* (2020 ▸)]. The eventual aim is to determine which symmetry property may explain the existence of flat bands in the electronic structure (Suarez Morell *et al.*, 2010 ▸): what, in the symmetry properties (if any) of twisted bilayers, is at the origin of this electronic localization?

A robust answer to this question requires a practical and simple crystallographic description of bilayer structures. This is the focus of the present work. The fundamental mathematical aspects of coincidence lattices at any dimensions are to be found in the very elaborated studies of Pleasants *et al.* (1996 ▸), Baake & Grimm (2006 ▸), Baake & Zeiner (2017 ▸). We focus here on the very elementary practical aspect of investigating the unique case of 2D bilayer structures.

Investigation of the symmetry properties of the superimposition of two 3D crystals, called bicrystals, was carried out in the 1980s (Gratias & Portier, 1982 ▸; Pond & Vlachavas, 1983 ▸) in the study of the properties of grain boundaries in metals and alloys. Although, at that time, these bicrystals were only theoretical concepts, their 2D versions of superimposing two monoatomic layers make sense in the present context as the idealization of a twisted bilayer considered as the superimposition of two infinitely thin monoatomic layers differently oriented by a twist rotation of angle α perpendicular to the layer plane and displaced with respect to each other by a translation τ in the plane.

The paper is organized as follows. Our first task is to enumerate which specific rotation angles α lead to a situation where two homophase layers share a common sublattice, say 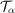 , of index Σ in Λ, and to explicitly give the expressions of these sublattices 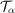 and those 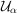 generated by the union of the lattices of the two layers. Our second task is to understand how these specific coincidence angles are distributed with respect to the values of the square length σ (identical to Σ for the square and hexagonal systems) of the coincidence unit-cell vectors. Our third task is to determine which space group 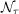 is generated for bilayers with coincidence lattices when the rigid-body translation τ varies at constant rotation α. Three appendices give the explicit illustration of the whole process in the case of twisted graphene bilayers and the conditions for coincidence and union lattices to exist in the case of heterophase bilayers obtained by dilatation and/or rotation or mechanical deformation.

We use the following notation: point groups are noted in capital letters like *G* or *W*; space groups and translation groups are noted in calligraphic letters like 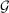 or 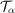 ; space symmetry operators (or functions in the complex plane as discussed next) are noted as 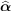 or 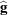 whereas point symmetry operators are simply written as α or *g*.

## 2. Elementary bicrystallography

As already mentioned, homophase bilayers are ideally defined here as the superimposition of two identical monolayers on top of each other, forming an infinitely thin layer of matter. The twist operation, that transforms the monolayer *I* into *II*, is either a rotation–translation 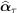 acting as 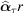 = 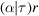 = 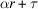 , or a mirror translation (in all 2D enantiomorphic structures, these two descriptions are equivalent as they describe the same twist operation) 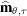 oriented along a direction of angle θ with the *x* axis, acting as 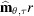 = 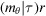 = 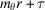 .

The original monolayer *I* has space group [we use the notations of Hahn (2005 ▸)] 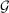 with point group Γ and lattice Λ showing the holohedral symmetry class of point group 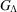 with 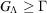 according to:

(i) Oblique system 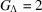 : 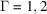 ;

(ii) Rectangular system 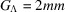 : 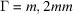 ;

(iii) Square system 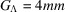 : 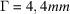 ;

(iv) Hexagonal system 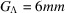 : 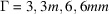 .

The corresponding group and lattice of the second monolayer *II* are given by 

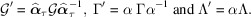

**Figure 1:**
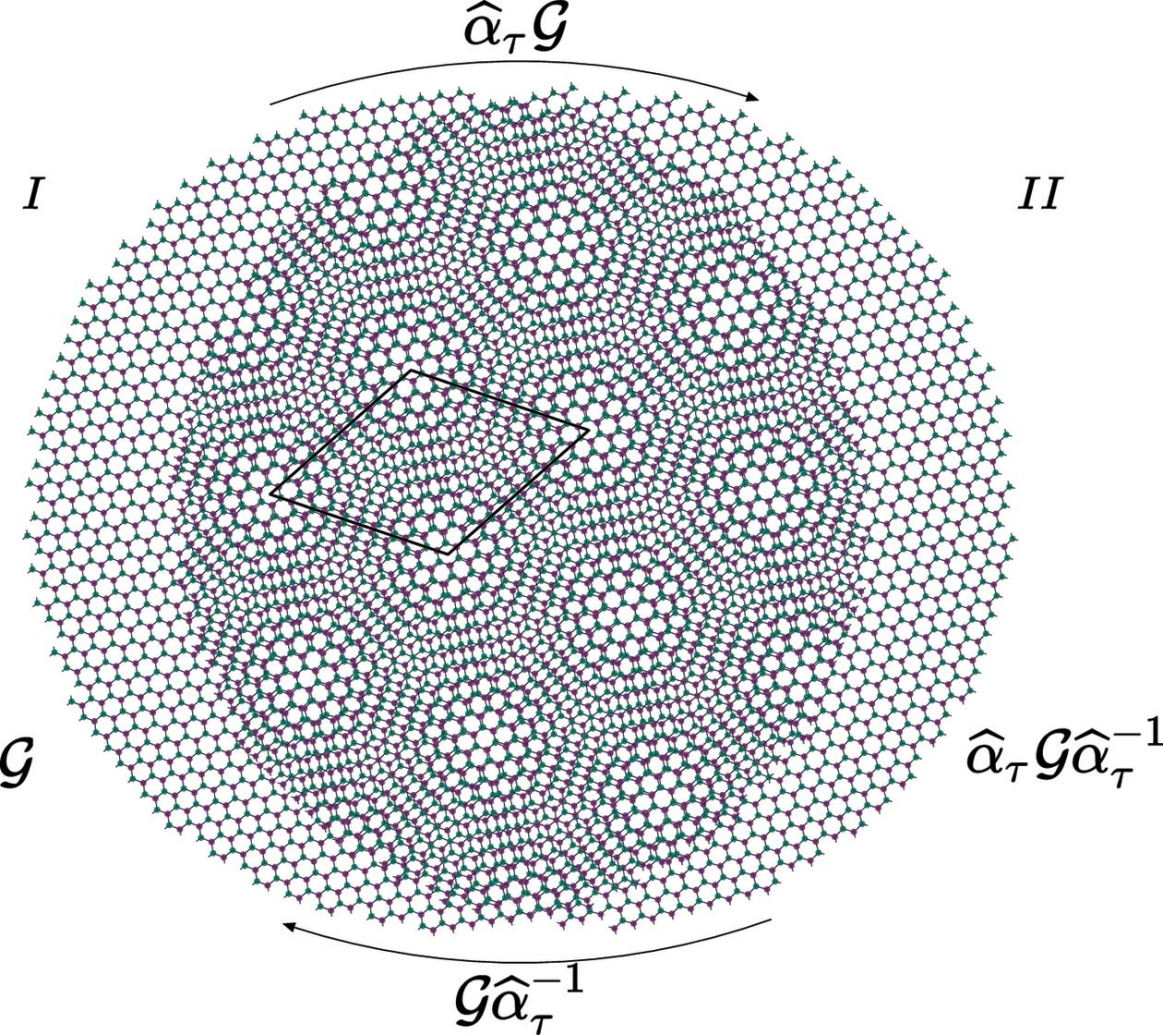
Passing from monolayer *I* to monolayer *II* is achieved by the set 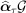 and from *II* to *I* by the inverse set 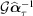 . The overlap of the monolayers, designed here as a bilayer, generates its own symmetry that is a 2D space group if the two lattices Λ and 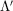 have a common coincidence lattice 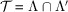 and only a quasiperiodic symmetry otherwise.

Since any point in the orbit of *r* under 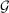 can be equivalently chosen, we characterize the transformation from layer *I* to *II* by the set 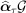 . The inverse transformation from *II* to *I* is given by 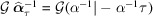 as shown in Fig. 1 ▸.

### 2.1. Using complex numbers for 2D crystallography

**Figure 2:**
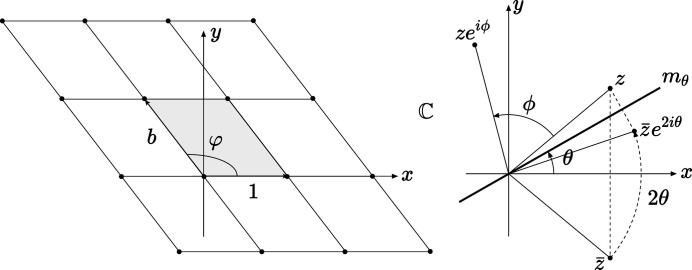
The lattice 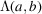 with unit cell 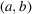 is the set of complex numbers 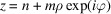 , 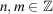 , 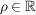 , where the unit vector *a* is chosen as the unity along the real axis *x* and *b* is the complex number 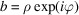 . A rotation of angle ϕ around the origin transforms *z* into 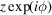 and a mirror 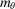 passing through the origin and oriented along the direction of angle θ transforms *z* into 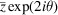 .

2D crystallography is particularly simple to handle using complex numbers. In fact, any 2D vector 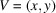 in an orthonormal reference frame of the plane is equivalently described by a complex number 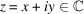 . Concerning the nodes of a 2D lattice 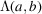 defined by its unit cell of vectors *a* and *b*, we choose the unit-cell vector *a* along the real axis and its length as the length unit with no loss of generality. The unit vector *b* is the complex number 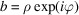 where ρ is the length of vector *b* in 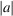 units and φ the angle of *b* with the real axis as shown in Fig. 2 ▸. A general primitive lattice 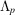 of unit vectors 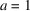 and 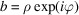 is then the set of complex numbers

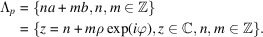

[In addition, *c*-type lattices encountered in the rectangular symmetry class ( 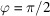 ) are defined as 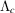 = 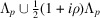 .] The complex notations of the 2D lattices are given in Table 1 ▸.

The symmetry operations act as functions of complex variable 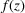 as elementary transformations of complex numbers:

(i) A translation 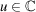 acts on a point *z* as 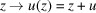 ;

(ii) A rotation ϕ around the origin transforms *z* into 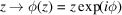 ;

(iii) A mirror 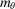 passing through the origin and oriented in the direction θ transforms *z* into 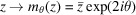 .

Space operators are the usual combinations of point symmetries and translations as shown in Table 2 ▸.

### 2.2. Coincidence angles for homophase bilayers

(This includes bilayers with different monolayers but sharing identical lattices.) General twisted bilayers are quasiperiodic structures built on a 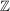 module of rank 4. Specific cases arise for particular values of the rotation angle α, called coincidence angles, where the two initial lattices Λ and 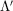 share a 2D sublattice 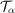 , called the coincidence lattice characterized by the index Σ [defined by equation (9)], the ratio of the unit-cell sizes of 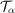 and 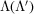 . This makes the nodes of the general 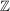 module of rank 4 condense on a 2D lattice 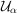 called the union lattice, discussed later, in a similar way to generating periodic approximants from quasicrystals. In fact, as will be shown next, coincidence angles occur an infinite countable number of times and form a uniformly dense set of values on the real axis: any generic twisted bilayer is infinitely close to a coincidence situation which is the only case leading to exact space symmetries of the bilayer.

Finding the proper coincidence angles has been the subject of a very large number of publications for 2D and 3D crystals (see, for instance, Ranganathan, 1966 ▸; Grimmer, 1973 ▸, 1974 ▸, 1984 ▸). The most complete and recent analysis of coincidence lattices in 2D crystals has been given by Romeu *et al.* (2012 ▸), a work that we reconsider here briefly using complex notations and that leads to a derivation which is simple and gives explicit expressions for the coincidence and union (homophase) lattices, as discussed next.

Let α be the rotation angle from the first monolayer to the second, both of point group *G*. The coincidence lattice, if any, is the common subset of the lattice translations of the monolayers: 

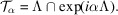

A first necessary condition for a coincidence lattice to possibly exist is that a lattice row defined by the node 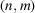 with 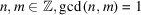 superimposes on another one 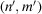 of the same orbit under 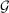 by the rotation α around the origin: 

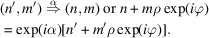

The possible generic solutions are listed below according to the crystalline system of the structure. {There are a few specific cases, in particular for the square system, with 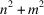 = 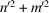 where the nodes 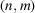 and 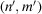 do not belong to the same orbit under 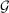 [for instance the nodes (3, 4) and (5, 0)]. These cases are not explicitly considered here.}

(i) Oblique system of point group 2: the generic orbit contains only two terms 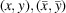 , so that there are no solutions but the trivial rotation 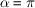 .

(ii) Rectangular system {this includes the special case of those specific oblique lattices where 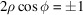 which should be considered as *c*-type rectangular lattices [ 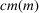 ]} point group 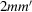 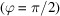 : the generic orbit contains four terms 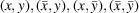 ; the two non-trivial solutions are those where 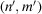 is deduced from 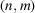 by the mirrors 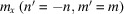 and 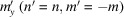 .

(iii) Square system of point group 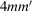 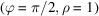 : in addition to the rectangle case, new solutions are 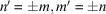 . All these solutions can be generated by using the mirror 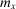 and the mirror 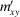 rotated by 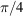 up to additional 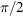 rotations.

(iv) Hexagonal system 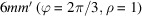 : here too, all possible solutions are obtained by using the mirror 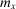 and the mirror 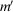 rotated from the *x* axis by 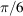 up to additional rotations of 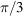 .

Hence, with the exception of the oblique system which presents no generic solutions, the rotation of a lattice node on top of one of its equivalents can be achieved using the two mirror generators of these point groups (see, for instance, Coxeter, 1963 ▸) according to: rectangle ( 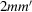 ) mirrors in the directions 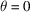 and 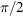 ; square ( 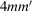 ) mirrors in the directions 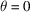 and 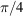 ; hexagonal ( 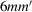 ) mirrors in the directions 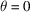 and 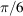 .

**Figure 3:**
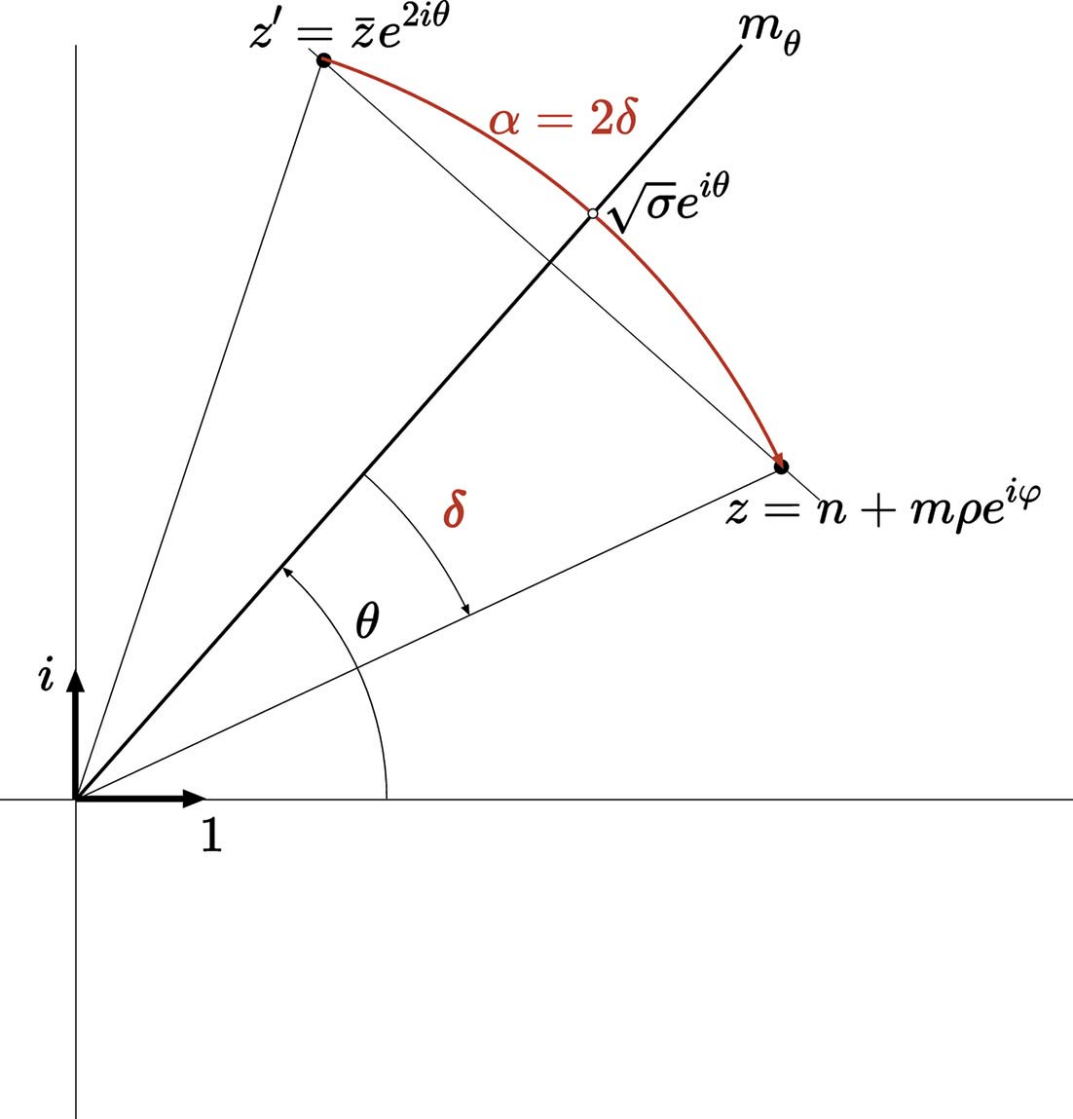
For all 2D systems except the oblique case, which has no generic solution, two homophase layers share the same crystallographic row defined by the lattice node 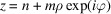 , with *n* and *m* coprimes, if there exists an equivalent lattice node 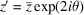 deduced from *z* by a mirror in the direction θ defined by: rectangle 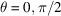 , square 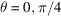 and hexagon 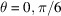 .

Let 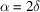 be the rotation around the origin that superimposes the node 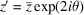 , on top of *z* = 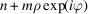 related to 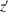 by the mirror 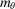 oriented along the direction θ as shown in Fig. 3 ▸. Putting 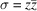 , we note that 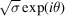 = 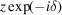 and therefore 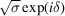 = 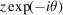 so that 

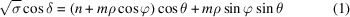




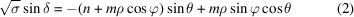

and thus

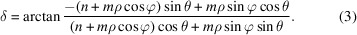

These three relations apply for the rectangle, square and hexagonal systems with the following specific forms:

Rectangle 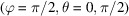 : 

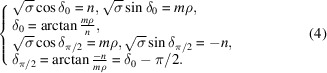




Square 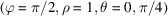 : 

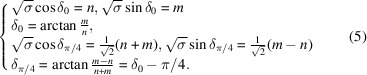




Hexagonal 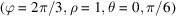 : 

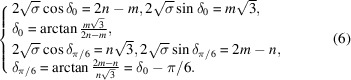




These relations are necessary conditions for ensuring two equivalent lattice rows superimpose on each other by the rotation 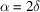 and 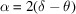 . Because these two solutions differ only by the constant rotation θ, we consider from now on the unique solution 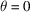 defined by the basic relations

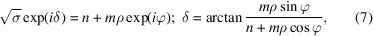

remembering that with each solution δ 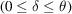 is associated the solution 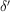 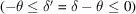 .

#### 2.2.1. Coincidence lattices in the rectangle system

Ensuring one row in coincidence is of course not sufficient to generate a 2D coincidence lattice: this requires another non-collinear row of lattice nodes to be in coincidence for the same rotation angle.

We discard the oblique system that we know has no generic rows of coincidence whatever the rotation angle and thus no possible coincidence lattice. We focus now on the unique rectangle system since the square and hexagonal systems are specific high-symmetry cases of the rectangle one.

Let 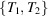 be the unit cell of the coincidence lattice 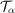 we seek with 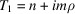 in the rectangle system. Because 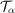 , if it exists, shares at least the same symmetry class as the lattice of the monolayer (see, for instance, Gratias & Portier, 1982 ▸) – here the rectangular symmetry 2*mm* or higher – another coincidence vector 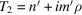 exists that is aligned along 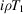 up to a certain ratio *r*: 

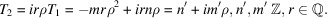

This requires 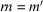 , thus 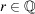 and 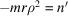 which is achieved if and only if 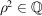 , * i.e.* 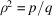 , where *p* and *q* are coprime positive integers. Thus, σ is a rational number: 

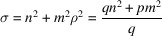

and 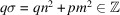 is a multiple of 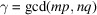 .

These results confirm in a few calculation steps those obtained by Romeu *et al.* (2012 ▸) following a seminal paper by Ranganathan (1966 ▸) in the context of classical 3D crystallography. Here, coincidence lattices in homophase bilayers in the rectangular system exist if and only if the ratio 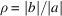 is the square root of a rational number: 

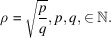

[Oblique lattices with 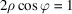 as the hexagonal lattice can be considered as rectangular *c*-type lattices of parameters 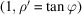 and therefore show a 2D coincidence lattice when 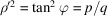 , with 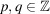 .] We conclude therefore that the coincidence angles for the rectangle system are distributed as a uniformly dense countable set of points on the real axis as 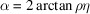 with 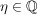 .

#### 2.2.2. Explicit expression of the coincidence lattice in the rectangle system

The unit vector 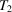 of 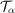 is the smallest vector along 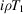 with integer coordinates

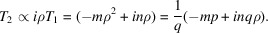

It is obtained by multiplying 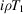 by *q* and then dividing the result by 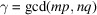 : 

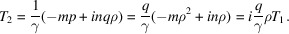

**Figure 4:**
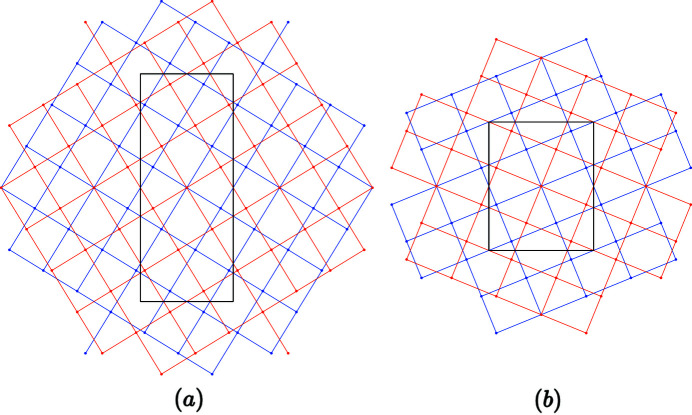
Example of coincidence lattices in black in the rectangular system 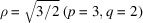 for: (*a*) *n* = 2, *m* = 1, γ = 1, 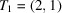 , 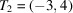 , 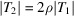 , 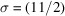 , Σ = 11, δ = 31.482°; (*b*) *n* = 1, *m* = 2, γ = 2, 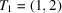 , 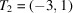 , 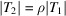 , σ = Σ = 7, δ = 67.792°.

We first note that putting 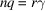 and 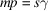 with 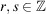 , we obtain 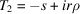 , which explicitly shows that, indeed, 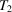 belongs to Λ. We then observe that, as required, 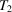 is orthogonal to 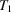 , but the length of 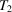 is in the ratio ρ with the length of 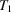 only when 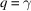 and therefore, although with at least the same symmetry class as Λ, the coincidence lattice is not necessarily homothetic to Λ in the general case as illustrated in Fig. 4 ▸.

Because of relations (7), we have

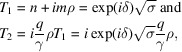

so that the coincidence lattice 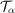 is explicitly given by

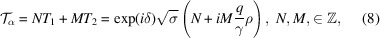

showing that the coincidence lattice 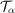 is generated by a lattice characterized by 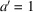 and 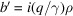 , rotated by δ with respect to Λ and linearly dilated by 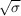 .

Since Σ is the index of the translation group 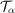 in Λ, *i.e.* the ratio of the surfaces of the unit cell of the coincidence lattice 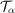 with respect to the one of the lattice Λ, we find

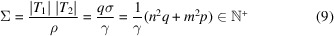

which is, indeed, an integer since 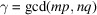 is a divisor of 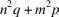 .

#### 2.2.3. The union lattice

The other fundamental translation group is the group 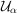 generated by the union of the lattice translation groups of the two crystals: 

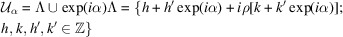

or 

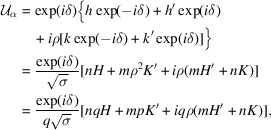

where 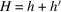 , 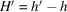 , 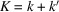 , 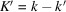 , 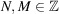 .

Therefore

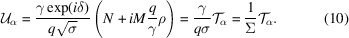

This shows that, for any coincidence angle and any symmetry class larger than or equal to the rectangular one, 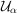 is homothetic to 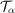 in the linear ratio 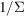 (this ratio applies on each unit vector leading thus to a relative density of nodes 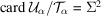 ). It is easily demonstrated that this relation holds for the square and hexagonal (see Appendix *A* [App appa]) systems with the coincidence lattices given by

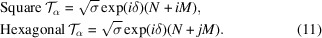




### Coincidence patterns 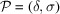


2.3.

**Figure 5:**
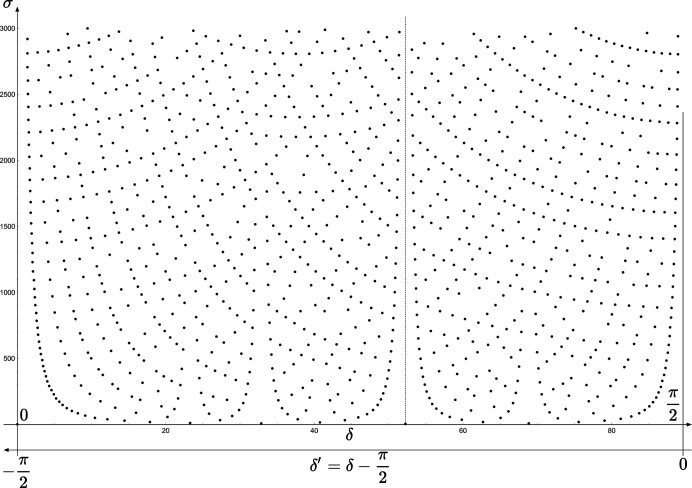
We visualize the set of coincidence angles in plotting the values of the coincidence angles 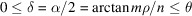 as a function of the square length 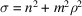 of the generating coincidence node 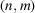 . Here, the example of the rectangle system with 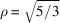 for 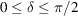 ; the other solution 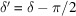 is obtained on the same diagram by exploring the *x* axis from 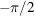 to 0. The vertical dashed line corresponds to 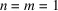 , *i.e.* 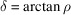 .

A classical scheme in metallurgy consists of collecting all the possible coincidence angles α, each associated with its Σ index, in a general pattern 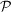 of points 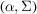 which is the superimposition of all the coincidence angles equivalent to α with respect to the intrinsic symmetries of the layer, each associated with its Σ. In the case of a rectangle system, this pattern can exhibit quite a complicated fine structure due to the arithmetic irregularities introduced by the term 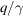 in the definition of Σ seen in equation (9). Moreover, this kind of pattern is heavily redundant because of the superimposition of several rotations that are equivalent with respect to the inner symmetry of the layer. In fact, as shown in Fig. 5 ▸, a simpler and equally informative pattern is obtained by plotting only one rotation representative in the elementary sector of the point group of the monolayer, as a function of the square length of the superposition node 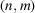 instead of Σ: 

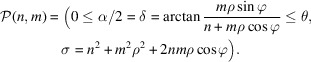

**Figure 6:**
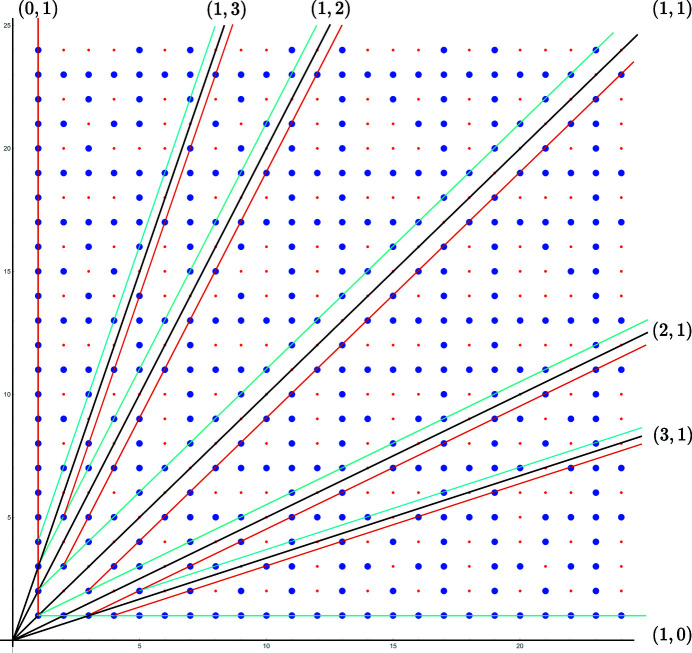
The points of coincidence are defined by coprime pairs of integers 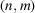 , *i.e.* by fractions 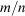 in their irreducible forms. Plotted on the nodes of a lattice, they generate the so-called set 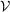 of points visible from the origin made of the lattice points drawn in blue. Each rational row of this set, as those drawn in colors, is associated with a branch of points with the same colors in the coincidence pattern of Fig. 7 ▸.

A very basic fact is that since the coincidence angles are defined by lattice vectors 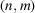 , where *n* and *m* are coprime integers, these vectors point to those nodes of a 2D lattice known as the set of points visible from the origin, noted here 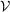 , as shown in Fig. 6 ▸. All points 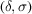 of the coincidence pattern 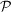 are in a one-to-one correspondence with those 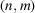 , of 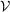 .

In particular, rational rows in the set 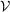 faithfully mirror the branches in 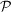 that are asymptotically converging to specific angles δ characterized by their coincidence nodes 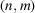 with 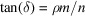 as exemplified by the rows and corresponding branches drawn in cyan and purple in Figs. 6 ▸ and 7 ▸.

The simplest way to classify and order these branches is to label them according to Farey sequences 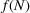 (see, for instance, Hardy & Wright, 1979 ▸). The Farey sequence of order *N*, noted 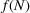 , is the set of fractions 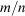 where *m* and *n* are coprime integers, associated with the nodes 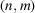 of the set 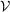 [see, for instance, in a different context Philippon (2008 ▸)], and such that 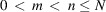 , ordered by size.

We note the following properties:

(i) For any two elements of a sequence, corresponding in the set 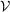 to the nodes 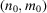 pointing in the direction 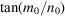 and 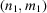 pointing in the direction 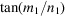 , with 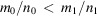 , the vector 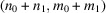 pointing along their diagonal is such that

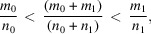

with 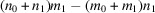 = 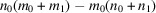 = 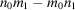 .

(ii) If two elements *i* and *j* are consecutive ( 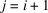 ) in a sequence with 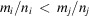 then 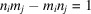 . Because of Bezout’s identity, we deduce that beyond 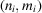 and 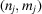 being coprimes, the pairs 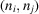 and 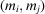 are also coprimes.

In fact, because the coincidence angles α run between 0 and π for the rectangle system, the sequences we are interested in here are extended Farey sequences (Halphn, 1877 ▸), noted 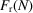 , made of the standard Farey sequence 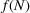 between (1, 0) and (1, 1) completed by the sequence from (1, 1) to (0, 1), obtained in adding to the original sequence the inverse fractions 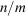 in opposite order. Such typical extended sequences for the rectangular system, where 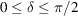 , are

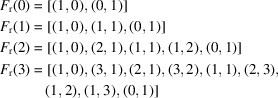


*etc*.

For the square system, the possible twist angles run from 0 to 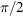 with the basic sequences ( 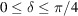 )

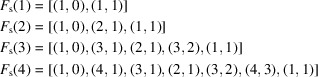


*etc*.

For the hexagonal system, with twist angles extending from 0 to 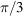 , the sequences ( 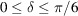 ) are

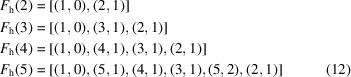


*etc*.

### 2.4. Invariance property of the branches

Defining branches of points in the coincidence pattern is pertinent when the points of the same branch, described by a running index *k*, share the same property independent of this index. To determine which invariance property a branch corresponds to, we note that, because of relations (1), (2) and (3), any two points 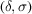 , associated with the coincidence node 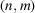 , and 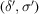 , associated with 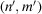 , of the same coincidence pattern are related by

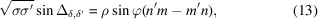

where 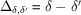 .

Relation (13) is the key for characterizing the invariance rule for each branch of the pattern.

We consider the case of the rectangle system ( 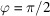 ) and choose two Farey neighbor terms 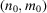 and 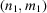 such that 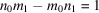 and 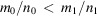 . We put 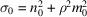 , 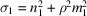 , 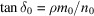 and 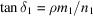 .

We consider the set of nodes

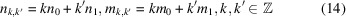

under their irreducible form [ 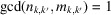 ], defining the points in 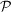 : 

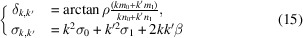

with 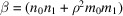 .

As shown in Fig. 6 ▸, at constant 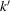 and running *k*, these nodes 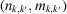 describe rows in 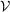 that are parallel to the direction 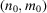 . At constant *k* and running 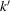 , they describe rows in the direction 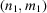 . These two rows intersect at the node 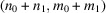 .

Observing that

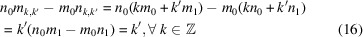




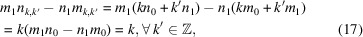

we note that at constant 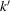 and running *k*, the points 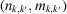 describe a set of branches in 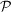 , one for each value of 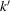 , asymptotic (by upper values for 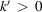 and by lower values for 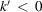 ) to 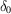 for 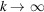 where all points share the invariance property: 

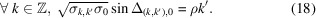




Similarly, from relation (17), at constant *k* and running 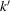 , corresponds a set of branches asymptotic (by upper values for 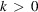 and by lower values for 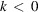 ) to 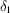 for 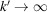 sharing the invariance property

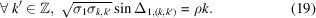

Concerning the irreducibility property, we note that 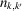 and 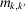 are both multiples of 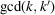 and therefore *k* and 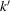 must be coprime for the node 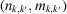 to belong to 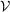 . Thus, any row in the set generated by a running 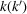 at constant 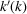 exhibits only the points that are not multiples of the prime factors of the constant 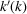 . For example, in the Farey sequence 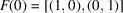 where 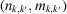 = 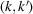 , the rows parallel to the *x* corresponding to running *k* at constant 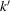 show, in increasing 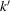 order: all *k* values for 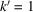 , only odd values of *k* for 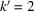 , *k* not a multiple of 3 for 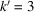 , *k* not a multiple of 2 and 3 for 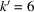 *etc*. The densest rows correspond to 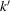 being a prime number. The same behavior is to be found for the rows parallel to the *y* direction and, extraordinarily enough, for any row parallel to a rational direction.

The 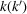 branches associated with the smallest values of 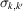 , designated here as optimal branches because they generate the smallest coincidence unit cell, are those where the constant 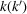 in relations (16) and (17) is the unity. These are the branches and associated rows colored, respectively, in cyan and purple in Figs. 6 ▸ and 7 ▸.

The two optimal branches in *k* and 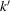 defined by the neighbor nodes 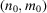 , 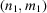 in the Farey sequence intersect at the node defined by 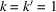 , *i.e.* at the node 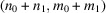 which is precisely the term inserted between the two original nodes in the Farey sequence next to the original one.

### 2.5. Analytical expression of the optimal branches

Although the coincidence angles form a dense enumerable set of points on the trigonometric circle, the proximity of two alpha values does not ensure that of their corresponding σ values. This happens only when the two angles are on the same branch. Two branches are particularly important which are asymptotic to the angles of the generating mirrors of the point symmetry of the lattice of the monolayer, *i.e.* 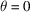 for all systems, with additional 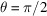 for a rectangle, 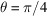 for a square and 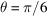 for a hexagonal system. They have a particular importance for bilayers with very small rotations as they allow us to choose the smallest-sized coincidence lattices closest to the angle we seek generating the smallest atomic model to be used in electronic calculations.

In the rectangle system, the two extreme asymptotic angles are 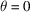 and 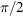 associated with the two extreme branches defined by the Farey sequence [ 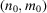 = (1, 0), 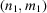 = (0, 1)]. The relation (4) leads to

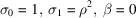




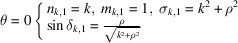




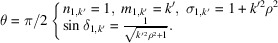

We assume 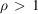 ( 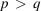 ). Using 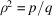 with 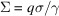 and 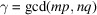 , we find

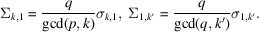

**Figure 8:**
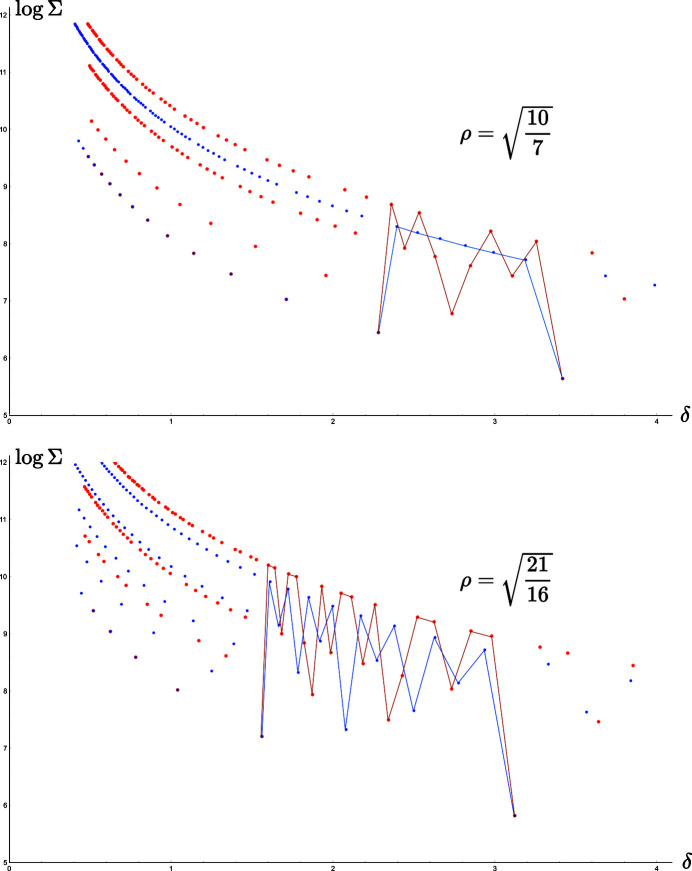
Splitting of the optimal branches as plotted against Σ instead of σ in the rectangle system for the cases 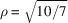 and 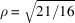 . The red dots and lines correspond to 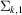 and the blue dots and lines to 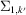 . In the first case, 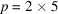 the red branch splits into four subbranches in the ratio 1, 2, 5 and 10, and since 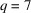 , the blue branch splits into two subbranches of ratio 1 and 7. In the second case, 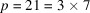 , the red branch splits into four subbranches in the ratio 1, 3, 7 and 21, whereas for 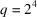 , the branch splits into five subbranches in the ratio 1, 2, 4, 8 and 16. In both cases, the red and blue branches superimpose for 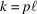 and 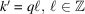 .

We first observe, as shown in Fig. 8 ▸, that each time *k* and *p* share the same divisor, 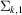 changes its value so that the initially unique 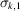 branch splits into ν subbranches 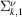 , where ν is the number of divisors of *p*. [Let 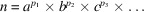 be the positive integer of prime factors *a*, *b*, *c*,…; the number of its divisors is 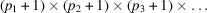 .] Similarly, 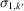 splits into μ subbranches 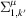 , where μ is the number of divisors of *q*.

We then note that the same angle δ is shared by the two branches at steps, respectively, *k* and 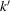 when 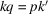 , * i.e.* for 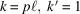 for one branch and 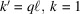 , for the other, 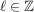 . At that stage 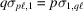 and therefore 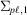 = 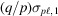 = 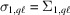 . The two branches superimpose every *p* steps for one branch and *q* steps for the other with the same Σ values. Hence, the optimal branch for small angles in the rectangle system ( 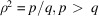 ) is found to be

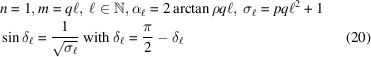

with a primitive lattice of parameters *A* = 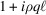 , *B* = 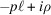 that condenses to a *c*-type lattice 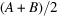 , 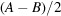 when *p*, *q* and 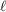 are all three simultaneously odd.

For the square system, the situation is much simpler since here 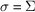 . The Farey sequence to be used here is 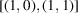 out of which we obtain

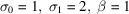




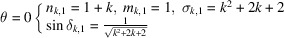




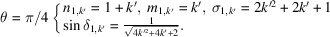

The same angle δ is shared by the two branches each time 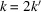 with, then, 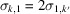 . This is easily understood by noting that 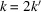 implies 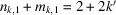 and 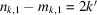 both even, which lead directly to 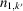 = 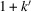 , 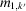 = 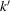 with a σ value twice smaller. The branch asymptotic to 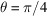 is therefore the optimal solution with the smallest unit cell defined by

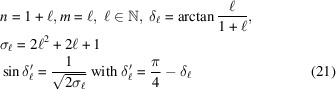

with a primitive lattice defined by 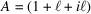 , 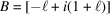 .

The case of the hexagonal system is treated in Appendix *A* [App appa] and leads to

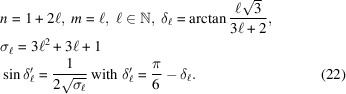

These results are easily understood by noting that the smallest coincidence angles are obtained when the two superimposed nodes are as close as possible to each other.

Indeed, applying relation (4) to the rectangle system with 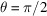 and 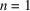 , 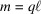 leads to

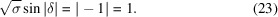

For the square system with 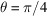 and 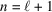 , 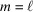 , relation (4) gives

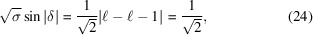

and for the hexagonal system, with 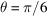 and 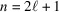 , 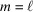




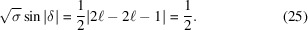




## 3. Space groups of homophase bilayers with coincidence lattices

**Figure 9:**
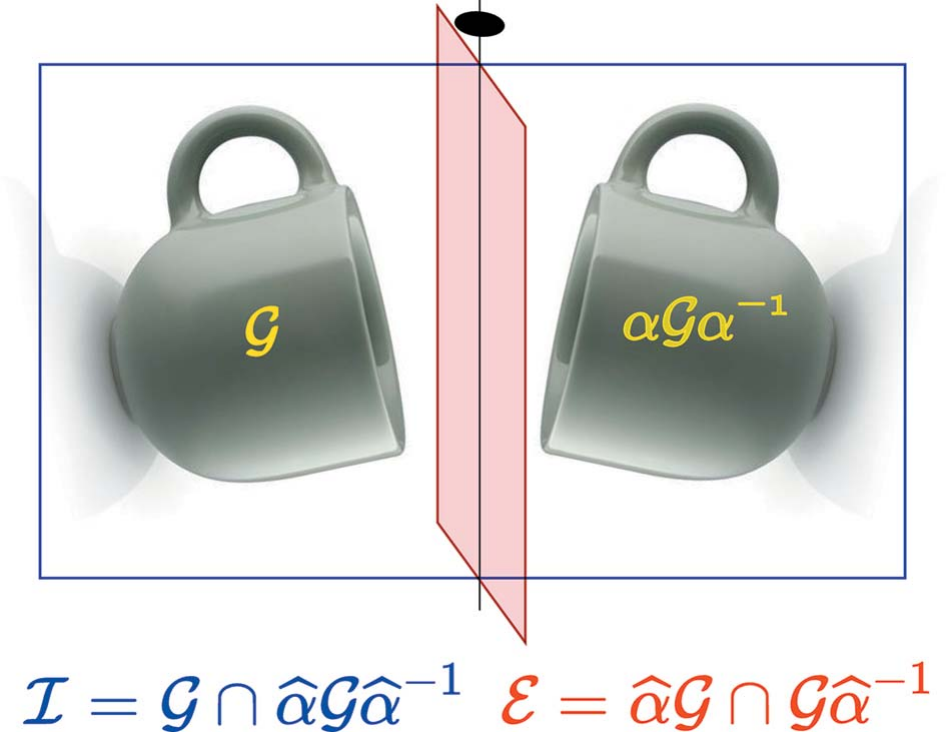
These two identical cups share the same mirror (blue frame) and transform into each other by another mirror (red frame) perpendicular to the previous one. Alone, each cup has the point symmetry *m*, but the pair of cups, taken as a whole, has point symmetry 2*mm*.

Building the space group of a homophase bilayer with a coincidence lattice is a simple work in principle that follows the same general scheme: the symmetry group of a set of two identical objects taken as a whole is the union of the symmetry elements that are common to both objects and are intrinsic symmetries of these objects plus extra elements, if any, that exchange the two objects as illustrated in Fig. 9 ▸.

It is easily demonstrated that the union of these two sets forms a group (see, for instance, Gratias & Quiquandon, 2020 ▸). Here, both the rotation α and the rigid-body translations τ are to be considered in the computations of these two basic sets:

(i) The intersection group contains those symmetry elements of the original layers that are of the same nature and superimpose in space,

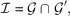

where 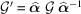 ; this group is never empty since it contains at least the identity and the translation group 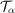 of the coincidence lattice.

(ii) The additional set of symmetry elements correspond to those extra new elements that exchange the two layers, transforming layer *I* into *II *and simultaneously *II* into *I*, defined by the intersection of the cosets 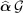 with 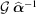 designated, if not empty, the exchange set 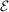 : 

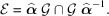

The symmetry group of the homophase bilayer, say 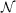 of translation group 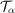 , is thus the union

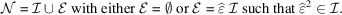




In addition to the group 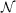 , another fundamental symmetry group of interest is the space group 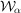 which generates, for a given rotation α and a given rigid-body translation τ, all translations 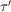 that generate equivalent bilayers, *i.e.* bilayers that can superimpose on top of each other by an isometry. Any two such translations τ and 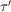 for the same rotation α are said to be equivalent. They form the orbit of τ in 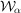 .

Because the rigid-body translation acts as a global translation of the layers, it is sufficient to consider only the orientational symmetry, *i.e.* the point group Γ, instead of the whole space group 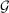 . The point group 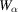 is obtained as in the preceding case, by considering the intersection of the point groups *I* and *II* and the exchange set but with the major change that, since the elements of the exchange set transform τ into its opposite, we must multiply the exchange set by the inversion operation: 

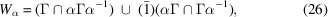

where 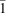 stands for the inversion operator. [A very unfortunate mistake is to be corrected in the work of Gratias & Quiquandon (2020 ▸) where the inversion operation has been forgotten in the expression of 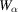 and improperly added in the one of 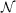 .] Here again, it is easily shown that 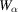 is a group.

The translation subgroup of 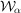 is found owing to the fact that adding to τ any translation of the lattices of either crystal transforms the bilayer into one of its equivalents. This translation group is the union group 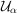 introduced in Section 2.2.3. The set of equivalent translations to τ is therefore the orbit of τ in the space group 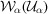 generated by the product of 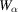 with the translation group 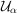 : 

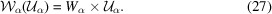

Hence, the number of different symmetry groups of the bilayers induced by varying the rigid-body translation τ for a given rotation α is the number of strata of the group 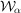 ( 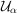 ). For example, as shown in Appendix *A* [App appa] and Fig. 13, there are only six different space groups for graphene bilayers whatever the rigid-body translation (and whatever the α rotation). It also shows that the natural reference frame to be used for labeling τ is the union lattice. Moreover, the domain of definition of the rigid-body translation τ becomes very narrow and decreases linearly as 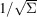 for coincidence angles α tending towards zero. As a consequence, at very small angles of rotation where the coincidence lattice unit cell increases dramatically, the unit cell of the union lattice becomes small enough for the rigid-body translation to become physically meaningless. Therefore, in the case of large moiré patterns due to small disorientations, it is not necessary to consider the rigid-body translation in the description (it can be chosen to be the null vector).

### 3.1. Finding the bilayer groups: point symmetry

The point symmetry elements to consider are rotations 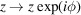 and mirrors 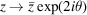 with 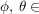 {0, 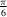 , 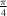 , 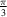 , 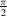 }.

The rotation α commutes with all the rotations of the lattice crystal since 

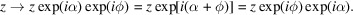

The intersection point group 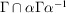 is thus the set of all the rotations of the point group of the crystal whatever the value of the coincidence angle α.

On the other hand, the exchange set 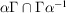 contains the mirrors generated by the product of the rotation α by the original mirrors, *i.e.* mirrors rotated by 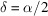 from the original ones. Indeed, the elements of 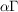 act on *z* as 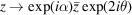 = 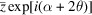 whereas those of 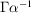 act as 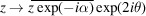 = 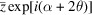 .

Therefore the exchange sets contain all the mirrors obtained by a rotation of 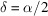 of the original mirrors of the structure whatever the value of the coincidence angle α. This explains why the coincidence lattice 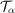 has the same point symmetry as the original symmetry class of the lattice. For the group 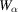 , the exchange set that contains all the mirrors is multiplied by the inversion, generating thus an equivalent set of mirrors but rotated by 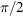 with respect to the initial ones.

### 3.2. Finding the bilayer groups: space symmetry

The space group 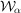 is easily determined since it is the direct product of 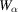 with 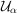 .

Concerning the group 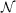 , the calculation requires a few steps.

Elements of 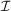 are the elements 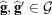 such that 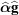 = 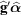 and elements of 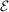 are the elements 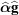 such that 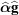 = 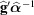 .

From 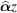 = 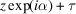 and 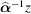 = 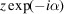 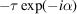 , with 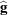 being either a rotation 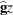 = 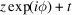 , or a mirror 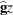 = 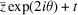 , we have the general explicit expressions

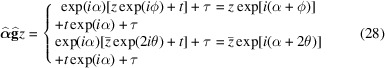




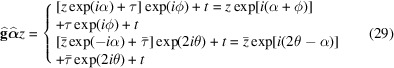




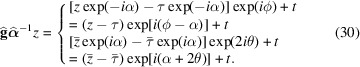

For an element being possibly included in either set 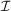 or 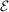 , the arguments of the variable *z* must be identical for the equalities to hold for any value of *z*.

Concerning 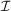 , the comparison between the lines (28) and (29) shows that the only possible solution for the elements 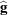 and 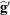 to be in 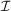 is two rotations of the same angle ϕ such that

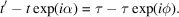

Concerning 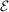 , the comparison of (28) with (30) shows that the pertinent elements 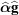 are obtained with 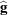 and 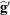 being parallel mirrors such that

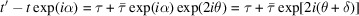

with 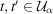 or possibly 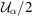 for the *c*-type space groups *cm* and *c*2*mm* and the non-symmorphic ones *pg*, *p*2*mg*, *p*2*gg* and *p*4*gm*.

Since 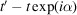 is a vector of 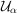 or 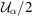 , we find that:

(i) The rotation 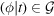 is in 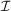 if τ is such that 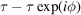 is a vector of 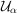 (or 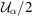 ) which is achieved for τ pointing to special positions of the group 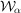 .

(ii) The mirror 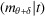 is in 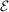 if τ is such that 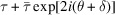 is a vector of 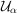 or 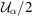 requiring thus τ to point along the perpendicular bisector of a mirror of 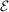 and thus τ to align along a mirror of 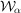 .

These two conditions lead to non-trivial solutions for τ being located at special positions of 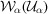 .

This shows that the space group 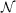 depends on the value of τ according to the different symmetry strata of 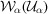 : the number of different possible space groups of the bilayer is equal to the number of symmetry strata of the group 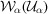 .

Moreover, the type of space group 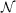 of the bilayer does not depend on the value of the coincidence angle α: whatever the value of α in that set is, the groups 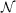 obtained for rigid-body translations τ with the same coordinates in 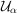 are isosymbolic; their actual representations in space are scaled according to the length 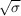 and the rotation 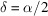 .

### 3.3. A simple low-symmetry example

**Figure 10:**
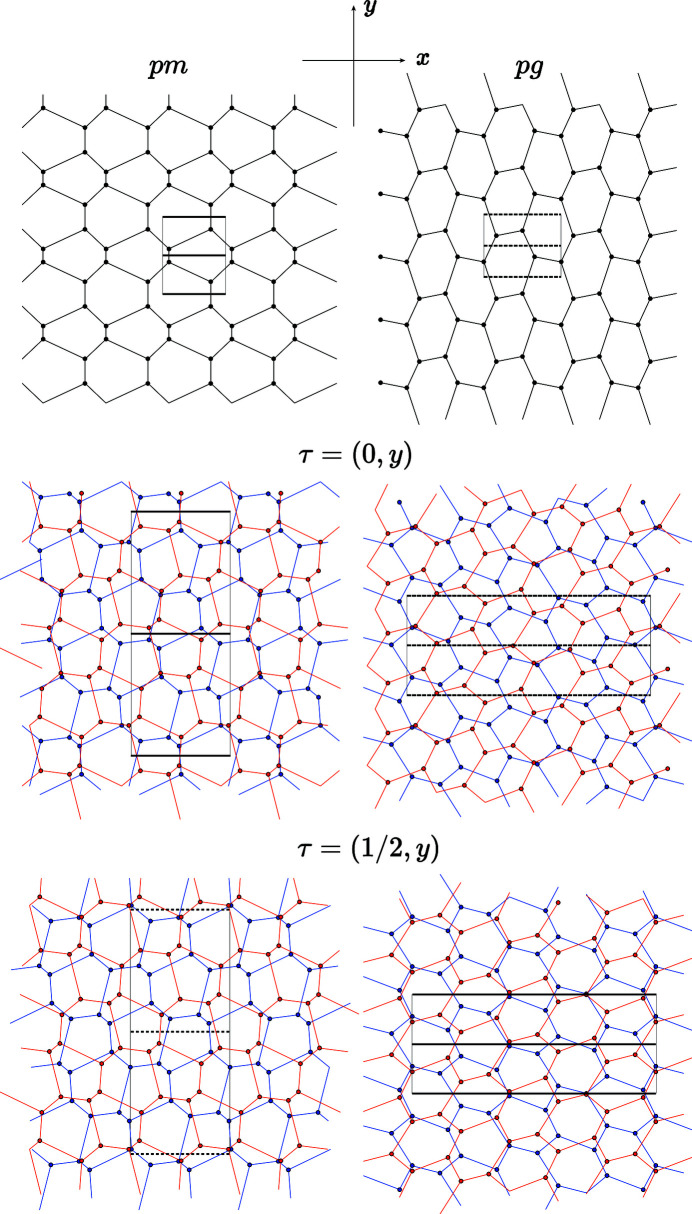
Example of structures of symmetry 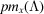 and 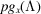 , showing that the mirror of the exchange set survives in the bicrystal symmetry only for the rigid-body translation τ located at special positions of the group 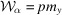 with translation group 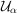 . Beyond the general position 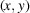 generating a bilayer of symmetry *p*1 and not shown here, there are two other strata 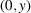 and 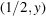 which generate a mirror in the bilayer structure. According to the values 0 or 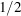 of the *x* component of τ on 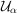 , these mirrors are either pure or glide. Here 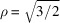 , *n* = 1, *m* = 1, α = 101.537°; the coincidence lattice 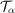 is defined by 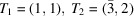 with 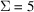 .

**Figure 13:**
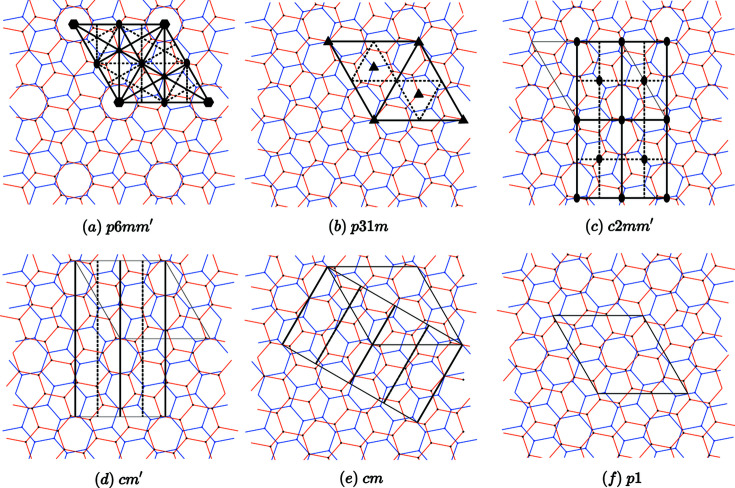
Example of a graphene bilayer with twist rotation 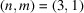 , 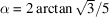 = 38.2132°, 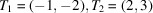 , Σ = 7, as a function of the rigid-body translation τ shown in Table 3. All coincidence angles α in 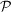 generate symmetry groups with the same symbols; they differ only by the scaling factor defined by the union and coincidence lattices.

We consider two bilayers *A* and *B* with coincidence lattices built from structures of symmetry class 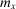 with space groups 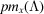 for *A* and 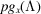 for *B*. In both cases, the point group 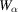 is made of the identity for the intersection group and 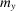 (original 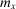 rotated by 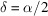 plus 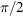 because of the inversion) for the exchange set: 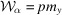 with translation group 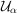 . This group has three strata expressed in the unit cell of 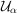 : 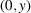 , 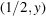 and 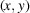 with little groups, respectively, 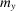 , 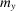 and 1. The translation 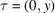 expressed in the unit cell of 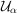 generates the group 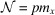 for the structure *A* and 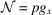 for structure *B*, both of translation group 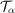 , and vice versa for the translation 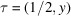 (Fig. 10 ▸).

## 4. Conclusion

To summarize, we find that infinitely many coincidence lattices generically exist down to the rectangle symmetry provided that the ratio ρ of the lengths of the unit-cell vectors is the square root of a rational number: 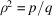 . They are generated by specific coincidence rotations of angle α of the form 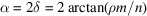 where *n* and *m* are coprime integers and can be written as

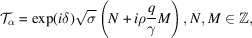

where 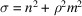 and 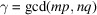 .

With each coincidence lattice is associated a union lattice 

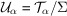

homothetic to 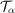 and which is the translation group of the space group of the equivalent translations of the rigid-body translation τ. Both coincidence and union lattices share at least the symmetry class of the original layer. For square and hexagonal systems, the three lattices 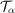 , Λ and 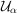 are two-by-two homothetic in the linear ratio 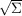 .

The complete set of possible coincidence lattices characterized by the rotation angle α and the unit-cell size Σ of the corresponding coincidence lattice form a diagram in one-to-one correspondence with the so-called set of points visible from the origin and can be analyzed using Farey sequences. They are distributed on branches, each characterized by a geometric invariant relating the sinus of the rotation angle to the square root of the unit-cell size.

In the case where a coincidence lattice exists, the space group of the bilayer depends on the value of the rigid-body translation τ between the two layers. There are as many different symbolic names of space groups as there are strata in the group 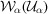 of the equivalent translations τ to a given one. These symbolic names do not depend on the value of the rotation α.

Because the group 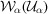 has 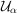 as translation subgroup, the unit-cell size of which tends to zero for rotations tending to zero, the rigid-body translation τ becomes a non-pertinent parameter – analogous to a phason field in quasicrystals – for twisted bilayers with very small rotation.

A subsequent work will discuss the case of general bilayers where 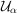 is a 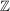 module of rank 4 in connection with the notion of 0-lattice which is independent of the possible existence of a coincidence lattice.

## Figures and Tables

**Figure 7:**
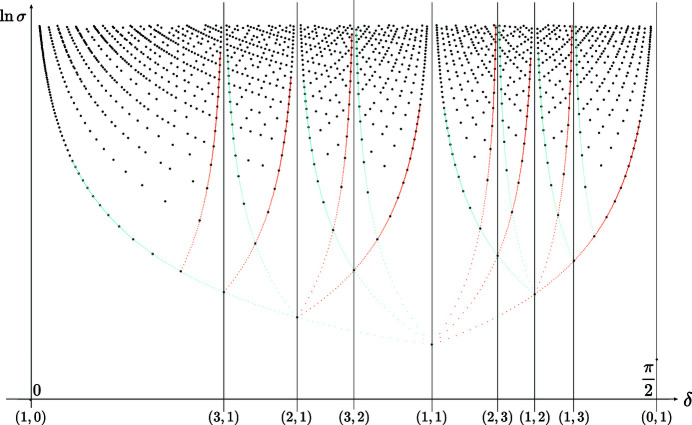
Distribution of the coincidence angles 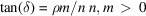 versus 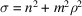 in logarithmic scale, for the rectangle lattice with 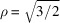 . The points are distributed on branches asymptotically converging to specific coincidence angles 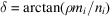 where the points 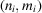 belong to (extended) Farey sequences generated from the initial pair 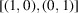 ; here, the optimum branches generated by the Farey sequence 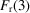 asymptotic by lower (purple) and upper (cyan) values are underlined with the same colors as their corresponding rows on the set 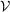 of Fig. 6 ▸.

**Figure 11:**
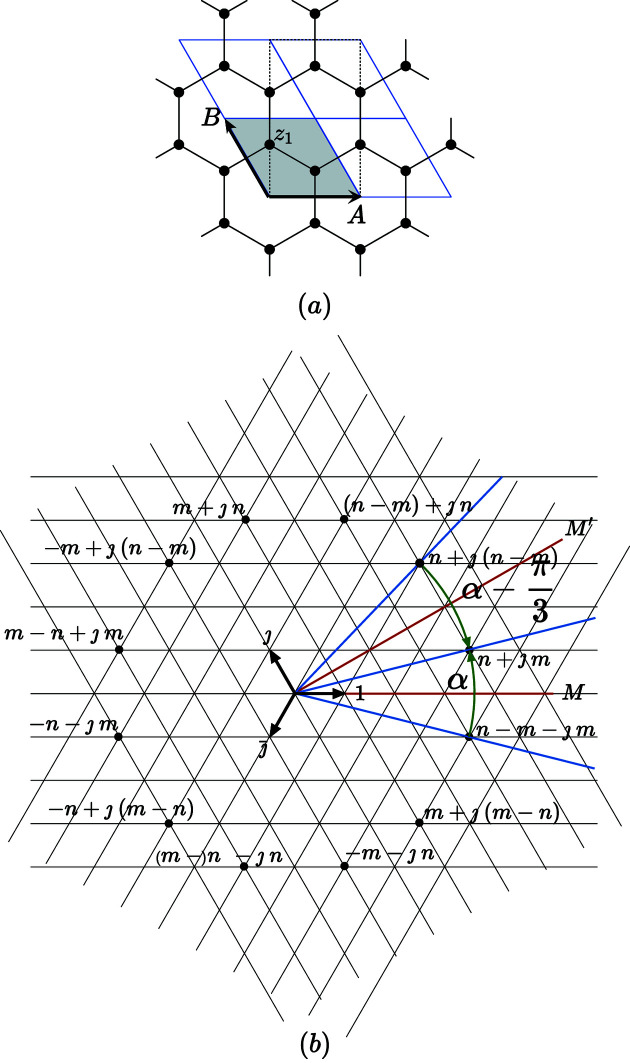
(*a*) Graphene is a 2D structure made of a honeycomb lattice of carbon atoms (in black on the picture). The standard primitive hexagonal lattice is generated by the pair 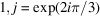 in complex notations defining the unit cell drawn in gray. The point symmetry group is 6*m* which can be generated by the two mirrors *M* and 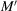 . (*b*) Generating coincidence lattices by rotation is easily obtained by applying rotations α around the origin that superpose nodes deduced from each other by the mirror along the real axis transforming the node 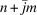 into 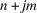 . Because of the hexagonal symmetry, choosing point 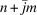 with 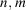 coprime in the region 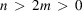 between the mirrors *M* and 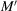 is sufficient for generating all the possible rotations of coincidence angles α and 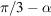 .

**Figure 12:**
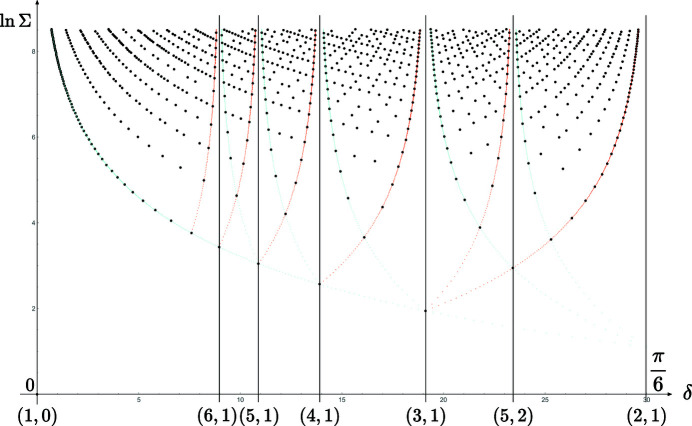
The rotations of coincidence 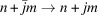 are defined by 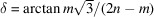 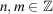 , 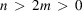 plotted for one unique twist rotation as a function of 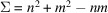 for 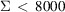 , on a logarithmic scale. As in the general case, well defined asymptotic branches are observed which correspond to the terms of the consecutive Farey sequences: the asymptotic branches of the 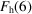 sequence are drawn in cyan and red.

**Figure 14:**
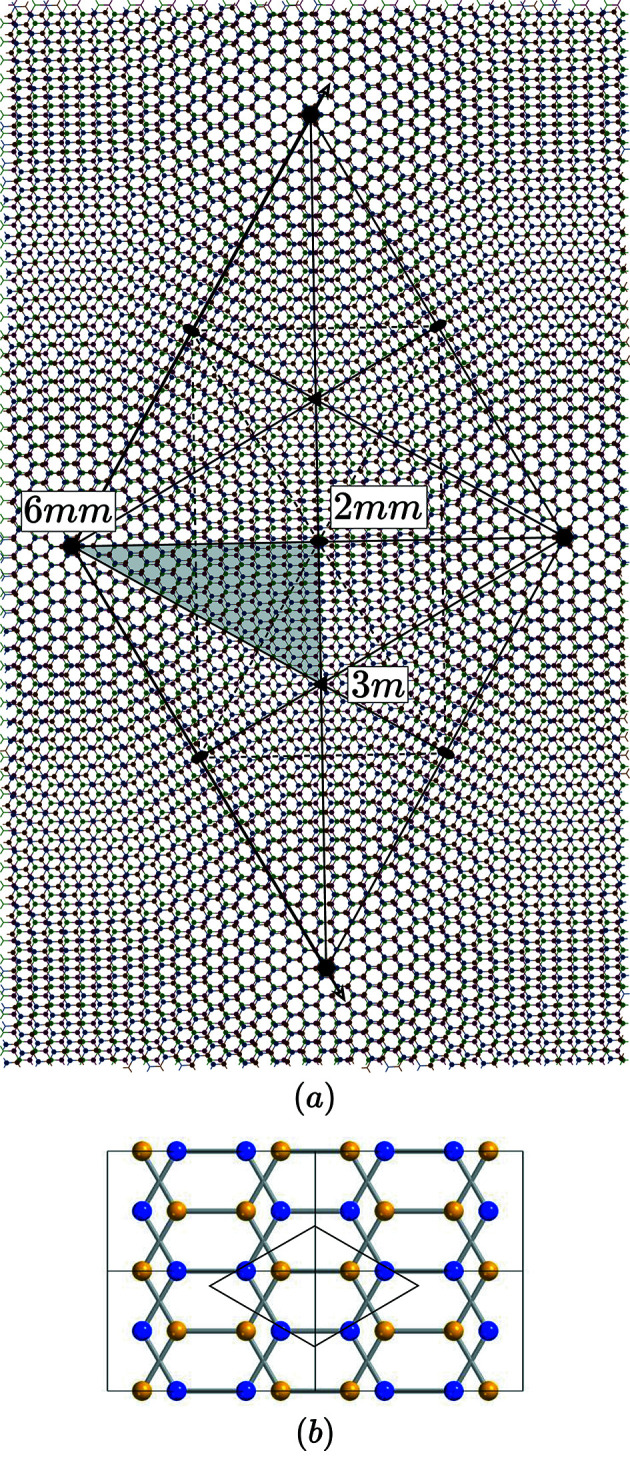
(*a*) Small rotations 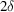 generate bilayers with the same symmetry as the original graphene layer, magnified by 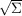 and rotated by δ. (*b*) Four unit cells of the local structure often called *SP* corresponding to the special point 2*mm*.

**Figure 15:**
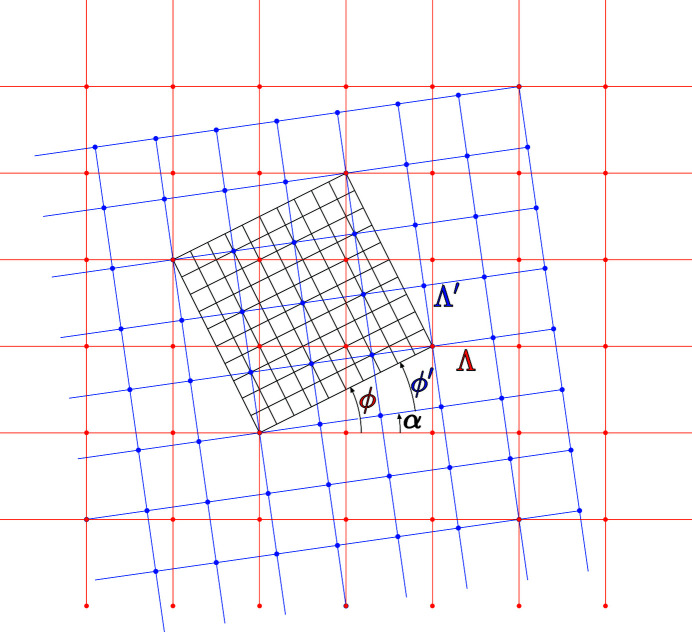
Example of a bilayer made of two square lattices related by a dilatation–rotation operation inducing a coincidence lattice: here the node 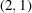 of Λ (in red) is superimposed on the node 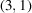 of 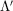 (in blue). The coincidence lattice is thus 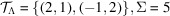 expressed in the unit cell of Λ or equivalently 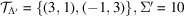 expressed in the unit cell of 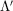 ; the dilatation is 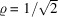 and the rotation 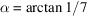 = 8.1301°.

**Figure 16:**
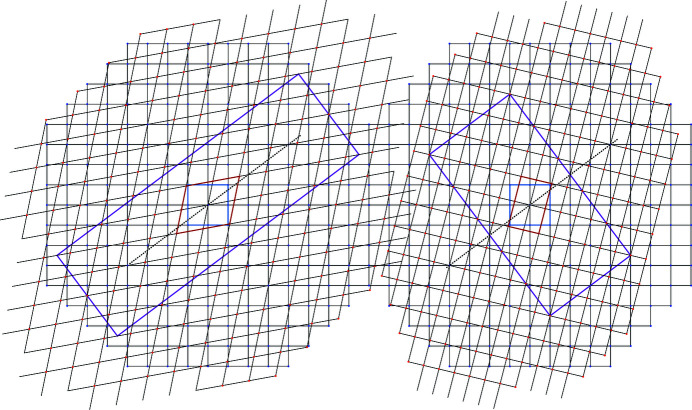
The square lattice in blue is transformed into the lattice in red after a rotation 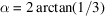 = 36.8699° (dashed lines) and (left) a dilatation 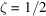 with coincidence unit cell: 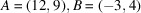 ; (right) a shear 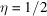 with coincidence unit cell: 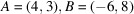 .

**Table 1:** 2D lattices: the parameter *a* is the length unit 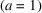 along the real axis All lattices are primitive except in the rectangular system with *c*-type lattices. Running indices 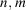 are integers. Here, 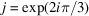 .

System	Lattice 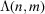	Unit cell 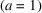	Angle 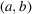
Oblique	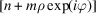	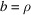	φ
Rectangular	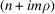	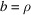	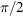
	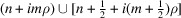	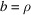	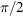
Square	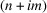	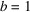	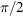
Hexagonal	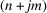	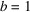	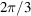

**Table 2:** 2D symmetry operations 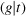 acting as functions 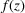 in the complex plane 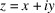

Symmetry operation	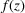	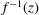
Translation 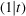	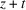	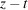
Inversion 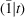	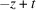	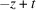
Rotation 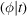	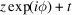	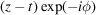
Mirror 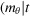 )	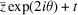	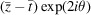

**Table 3:** Symmetry groups 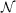 of graphene twisted bilayers as a function of the rigid-body translation τ expressed on the basis of the union group 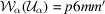 with origin chosen on the sixfold axis Its translation group is the union lattice 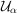 defined by 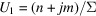 and 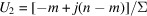 with 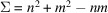 .

	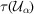	Little group in 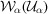	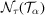	Label in Fig. 13
1*a*	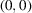	6*mm*′	*p*6*mm*′	(*a*)
2*b*	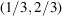 †	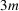 †	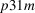 †	(*b*)
3*c*	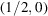	2*mm*′	*c*2*mm*′	(*c*)
6*d*	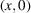	*.m*′	*cm*′	(*d*)
6*e*	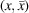	*m.*	*cm*	(*e*)
12*f*	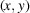	1	*p*1	(*f*)

† The rigid-body translation 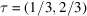 corresponds to the natural stacking in graphite.

## Appendix A The specific example of graphene bilayers

Graphene has a 2D periodic structure of group  with a carbon atom at special position  . It is described in the complex plane  by the primitive hexagonal lattice Λ (  ) defined by

with a carbon atom at position  [and equivalently  ] as shown in Fig. 11 ▸(*a*). [It turns out that the commonly used notation in the physics community of graphene is to take the hexagonal reference frame with the acute angle  instead of the crystallographic definition that uses the angle  . This corresponds to choosing  as reference frame instead of  . Noting thus that a node *z* can be equivalently written as  we obtain  and  and thus  .] The unit-cell parameter equal to *a* = 0.2456 nm is chosen here as the unit length.

The point symmetry elements of 6*mm* are generated by the rotation of  located at the origin and transforming (the six hexagonal rotations are  ) *z* into  and the mirror along *x* transforming *z* into  . The orbit  of a generic point *z* has thus 12 elements per unit cell:

as exemplified in Fig. 11 ▸(*b*).

### A1. Graphene bilayers with coincidence lattices

Twisted graphene bilayers are certainly among the most studied materials in the world [see, for instance, the recent review by Geim (2009 ▸)], often created, for example, in epitaxial graphene growth on the C-terminated face of Si–C (see Campanera *et al.*, 2007 ▸; Hass *et al.*, 2008 ▸; Varchon *et al.*, 2008 ▸; Bistritzer & MacDonald, 2011 ▸). These twisted bilayers are the superimposition of two single graphene sheets slightly twisted with respect to each other by a small angle α of a few degrees or less. It was seen a couple of years ago that these twisted graphene bilayers have remarkable electronic structures (see Trambly de Laissardière *et al.*, 2010 ▸). As already mentioned, from the geometric point of view discussed here, the two graphene sheets are considered as infinitely thin and located on the same plane.

Twist rotations of angle α leading to coincidence lattices are infinitely many (see Feuerbacher, 2021 ▸). They are characterized by the rotations α that superpose a representative of a given orbit of nodes  on top of another point of the same orbit  . As previously mentioned, because of the high symmetry of the hexagonal system, it is enough to examine the rotation  around the origin that transforms the lattice point  into  as shown in Fig. 11 ▸, where *n* and *m* are positive coprime integers with  :

or

where  and  , as application of equation (7) to the hexagonal system.

### A2. Hexagonal and rectangular coordinates

The connection between the rectangular *c*-type lattice with  reference frame with coordinates  both integers or both half-integers and the hexagonal lattice reference frame  both integers is given by

It is easily verified that σ is an integer for both  and  being half-integers,

and that

{The relation  , found for the rectangular system, is based on  =  =  and leads to  – and thus  as expected – when  is not a multiple of 3, but to  when  and thus  which seems contradictory to the present result. In fact, because  , the sum  is a multiple of 3 and the actual coincidence unit cell reduces to  , which is indeed three times smaller.}

### A3. Coincidence and union lattices

Relation (6) leads to

and

The unit vectors of  are  and  =  =  ; because of relation (33), this translates into  and since  :

The coincidence lattice  is an hexagonal lattice deduced from the original lattice Λ of the layer by a rotation of δ with unit-cell parameter  . The calculation of the union lattice  leads to the same expression as in equation (10):

which is the original hexagonal lattice rotated by δ with a unit-cell parameter linearly shrunk by  .

### A4. The coincidence pattern

The coincidence pattern is shown in Fig. 12 ▸, generated by one unique representative out of the 12 equivalents of the rotation, with the corresponding Σ plotted on a logarithmic scale. As already discussed in Section 2.3, the coincidence points are distributed on branches converging asymptotically to specific rotation values when  . Putting  =  and  from equation (14), we obtain

with  . The basic invariance relations (13) for each branch are in hexagonal coordinates:

where Cte stands for a constant value. Here, again, the optimal branches are those where  for running  drawn in cyan and red in Fig. 12 ▸. Of greatest importance are the optimal branches associated with the nodes of the initial Farey sequence  since they cover the entire angular definition domain of  generating the smallest Σ values. We find

The same δ angle is found between the two branches for  with  . Indeed, since then  =  we see that  =  and, of course,  =  are both multiples of 3, leading to a unit cell three times smaller. This shows that the asymptotic branch  is the optimal solution leading to the smallest unit cells:

### A5. Symmetry groups of twisted graphene bilayers

Our last task is to analyze the overall symmetry of the graphene twisted bilayers with coincidence lattices. The group  is easily found as  of translation group  whatever the value of the coincidence angle δ in  . The group *p*6*mm* contains six symmetry strata listed in Table 3 ▸. There are thus only six different possible space groups for the bilayer according to the coordinates of τ expressed in units of the union lattice  as shown in Table 3 ▸ and exemplified in Fig. 13 ▸.

The case of very small rotations deserves some attention. Rotations decreasing to zero are associated with coincidence lattices with larger and larger unit cells and therefore to shorter unit cells for the union lattices. As noticed in the body of the text, this leads to rigid-body translations tending to zero and therefore losing any physical pertinence. Small rotations can indeed be locally described as translations between the two almost-parallel layers, as shown in Fig. 14 ▸(*a*). The normalizer at  is the group of the graphene layer, *p*6*mm*, scaled by  and rotated by δ: the bilayer has exactly the same symmetry properties as the initial graphene unit cell but magnified to mesoscopic scales. It can be roughly described as an hexagonal tiling made of three main microscopic high-symmetry structures occurring at each special point of the large hexagonal coincidence unit. In that renormalization-like view, the centers of the initial hexagons are replaced by the structure of local symmetry 6*mm*, usually designated as *AA*, that is the graphene itself, the carbon atoms by the structure of local symmetry 3*m*, called  , as in the natural graphite, and the binding between carbon atoms, designated as *SP*, by the structure 2*mm* shown in Fig. 14 ▸(*b*) of space group  with cell parameters  and two carbon atoms at positions  and  . These three basic high-symmetry structures, 6*mm*, 3*m* and 2*mm*, correspond to the first three special positions of dimension 0 as shown in Table 3 ▸ and Fig. 13 ▸. This exhausts all the symmetry possibilities: there are no other kinds of structures, for any  and any (here, meaningless) values of τ.

## Appendix B Heterophase bilayers with coincidence lattices

### b1. Homogeneous dilatation–rotation coincidence lattices for heterophase bilayers

Heterophase bilayers are formed by two layers of different structures; they show very similar geometrical properties to the homophase bilayers. However, our present context of using complex numbers allows us to treat here only those heterophase bilayers where the lattices Λ and  of the layers can be deduced from each other by a dilatation–rotation, *i.e.* when

Here,  is the dilatation coefficient and α the rotation from Λ to  . This kind of transformation is the general case when the lattices belong both to either the square or the hexagonal systems as examplified in Fig. 15 ▸.

We choose here to discuss heterophase bilayers in the square system for simplicity.

Let Λ and  be the two square lattices of lattice parameters  for Λ and  for  :

A coincidence lattice  exists if two pairs of integers  and  exist with  such that

in which case the coincidence lattice is  ,  expressed on the unit cell of Λ or equivalently  ,  expressed on the unit cell of  . Explicitly

or by putting  ,  , we obtain

Therefore, two square lattices (it can be easily verified that the same property applies for the case of hexagonal lattices) of different sizes can share a coincidence lattice only if the ratio of the unit-cell lengths is the square root of a rational number  in which case the area of the coincidence unit cell is simultaneously an integer multiple Σ of the area of the unit cell of the first lattice and another integer multiple,  , of the unit cell of the second lattice in the ratio of the rational number  .

Relation (37) leads to

Let ϕ and  be the rotation angles between the unit cell of the coincidence lattice  and those of, respectively, Λ and  (see Fig. 15 ▸); we have

and therefore

Also, because  :

or

which is consistent with the expression of α given by relation (38).

The union lattice  is given by

It is easily checked that, in the homophase case, where  ,  , this relation simplifies to (10).

Fig. 15 ▸ gives a simple example of a rotation–dilatation transformation between two square lattices  where the node  of Λ (in red) superimposes on the node  of  (in blue) by a rotation α and a dilatation  . Here  and  . Thus, we find  and  . We have  with  ,  and  with  and  .

#### b1.1. Pure dilatation

A pure dilatation of one layer with respect to the other is characterized by  and therefore  or  . We assume *n* and  are non-zero, leading to

The union lattice  is written

## Appendix C Homophase bilayer under mechanical deformation

The two layers can in general be of two different structures of space groups  and  with lattices, respectively, Λ and  . We still designate by  the transformation from Λ to  ,

as exemplified in Fig. 16 ▸. For simplicity, we treat here the case of the initial lattice Λ belonging to the square system.

### c1. Pure shear deformation

Here  results from a pure shear deformation of the square lattice Λ of parameter  in the direction of angle α with the *x* axis of Λ and of intensity η:

with the shear direction  .

If we choose: (i) the angle α among those generating a coincidence lattice,

as discussed in the body of this article, using  , and

(ii) the shear intensity η rational with respect to the square lattice parameter

then the transformation  is written as a matrix with rational coefficients,

which generates a coincidence lattice defined by the unit vectors

where  .

### c2. 1D dilatation

A dilatation of the square lattice Λ of intensity ζ in the direction of angle α with respect to the *x* axis of Λ can be written as

As in the previous case, if  and  , the transformation  is a matrix with rational coefficient

with lattice parameters

where  .
